# Motivating Social Influencers to Engage in Health Behavior Interventions

**DOI:** 10.3389/fpsyg.2022.885688

**Published:** 2022-07-22

**Authors:** Crystal R. Smit, Kirsten E. Bevelander, Rebecca N. H. de Leeuw, Moniek Buijzen

**Affiliations:** ^1^Erasmus School of Social and Behavioural Sciences, Erasmus University Rotterdam, Rotterdam, Netherlands; ^2^Radboud Institute for Health Sciences, Radboud University and Medical Centre, Nijmegen, Netherlands; ^3^Behavioural Science Institute, Radboud University, Nijmegen, Netherlands

**Keywords:** social influencers, health behavior, interventions, youth, motivation

## Abstract

Social influencers are widely known as the promotors of purchase behavior as well as for their potential to change health behaviors among individuals in their social networks. For social influencers to be successful in changing behaviors, it is essential that they convey their message in an authentic, original, credible, and persistent manner. In the context of health behavior interventions, this requires a focus on the motivation of social influencers to engage in the intervention. This perspective article describes the importance of motivating social influencers to engage in the desired health behaviors themselves and to promote it within their social network. We briefly describe the current state of knowledge and our empirical experience in implementing health interventions with social influencers. Using insights from self-determination theory, we demonstrate how social influencers can be motivated optimally in health behavior interventions and, thereby, improving the success of the intervention. To illustrate these insights and guide intervention practice, we provide concrete examples of techniques that can be applied in health interventions involving social influencers. We conclude with directions for further research and intervention practice to improve the delivery of health behavior interventions.

## Introduction

Social influencers are widely known as the promotors of (purchase) behaviors to influence individuals in their social networks for marketing and advertising purposes [for a review, see Vrontis et al. (2021)]. They can also effectively promote and change health behaviors within their social networks to prevent communicable and non-communicable diseases [for a review, see Hunter et al. (2019)]. The role in which they exert their influence can range from a popular influencer on social media to a natural peer influencer in a school classroom (Hunter et al., 2019; Vrontis et al., 2021). For a successful development of campaigns involving social influencers, it is essential that they can convey their message in an authentic, original, credible, and persistent manner (Santiago and Castelo, 2020). Recent empirical research on social influencers has also shown that perceived credibility and authenticity of influencers by their followers are important indicators of campaign success (e.g., Munnukka et al., 2019; Pöyry et al., 2019; Reinikainen et al., 2020; Pittman et al., 2022). To achieve this, campaign developers must ensure that social influencers are motivated to be engaged in the campaign and the related behaviors (Lin et al., 2018; Audrezeta et al., 2020).

A recent practical example that highlights the relevance of this, is the COVID-19 “#alleensamen” [#onlytogether] health campaign of the Ministry of Health, Welfare and Sport in the Netherlands. In this campaign, social influencers were rewarded extrinsically with money to post a video or message on their social media to promote the government-imposed COVID-19 measures on social distancing within their social networks. A few months later, some of these influencers turned against the government policy and urged others on social media using the #ikdoenietmeermee [#idonotparticipateanymore] hashtag to stop adhering to these COVID-19 measures (Nederlandse Omroep Stichting NOS, 2020). This could possibly have been prevented if more attention had been directed to the intrinsic motivation of these social influencers to promote COVID-19 measures. Therefore, in this perspective article, we discuss the importance of focusing on the intrinsic motivation of social influencers to engage in health behavior interventions, thereby allowing them to be perceived as credible and authentic by their followers.

Social influencers have been extensively included by marketing and advertising industries in their campaigns to influence the purchase behaviors of individuals in their social networks (De Veirman et al., 2019; Vrontis et al., 2021). A well-known promotional activity of social influencers in marketing and advertising is the so-called *unboxing* videos in which they unpack a product, try it out, and share their experiences on their social media channel. Products endorsed by these social influencers include, for example, toys (Craig and Cunningham, 2017), makeup (Wiedmann et al., 2010), and predominantly unhealthy foods and beverages (Coates et al., 2019; Smit et al., 2020). During the past years, there has been a growing interest in implementing this social influencer strategy to promote health behaviors. There has been ample research conducted on so-called “social-network interventions” in which the most central individuals in the network are identified as influencers to promote health behavior change within their social network (Valente, 2010). Most of these interventions involving social influencers to promote health behavior change have proven successful, however, there are a few examples of interventions that were not successful (Bell et al., 2017; van Woudenberg et al., 2018, 2020). Social influencers proved successful in promoting condom use among gay communities (Kelly et al., 1997; Amirkhanian et al., 2015), smoking cessation among young adolescents (Valente et al., 2003; Campbell et al., 2008), water consumption among children (Smit et al., 2016, 2021a; Franken et al., 2018), and physical activity among young adolescent girls (Sebire et al., 2018). In addition to these offline social influencer examples involving real-life connections, attention has recently been growing for the use of social influencers to promote health behaviors in online social networks through social media. For example, social influencers have effectively promoted healthy snacking (Folkvord et al., 2020) and flu vaccination (Bonnevie et al., 2020) among their online followers.

There are various motivations for popular influencers on social media as well as natural peer influencers in classrooms, schools, and communities to engage in campaigns, ranging from extrinsic to intrinsic motivation. For example, extrinsic motivation involves social influencers being paid or compensated with free products to promote the behavior, while an intrinsic motivation could involve their deep interest or enjoyment of the related product or service (Lin et al., 2018; Audrezeta et al., 2020). Many social influencers have begun to share behaviors within their social networks based on an intrinsic motivation, for example, as an outlet for themselves or because they enjoy inspiring, helping, or educating others. As their audience grows, their motivations may also gradually change to or expand with extrinsic motivation when they promote (purchase) behavior for money and free products (Audrezeta et al., 2020). However, some influencers perceive that with sponsored content, the post is not as popular among their followers compared to a non-paid post (Neal, 2017). As a result, they can become less motivated when it comes to creating and sharing sponsored posts and behaviors (Neal, 2017; Audrezeta et al., 2020). This emphasizes the importance of focusing on intrinsic motivation in interventions with social influencers to keep them engaged in the promoted behaviors. Additionally, research focused on health behavior change shows that individuals who are intrinsically motivated are more likely to adopt and maintain health-related behaviors over time (Silva et al., 2011; Hagger et al., 2014; Smit et al., 2018). Therefore, it is especially important when the intervention targets health behaviors to focus on enhancing the intrinsic motivation of social influencers. In the next section of this article, we briefly describe the current state of knowledge on motivating social influencers to engage in health behavior interventions.

## Motivating Social Influencers

Lately, there has been a growing awareness of the importance of training social influencers to convey the desired health behaviors [for a review, see St. Pierre et al., 2021]. Researchers suggest that thorough training of those who deliver the message is key to a successful health intervention (Langford et al., 2015; van Woudenberg et al., 2018; Smit et al., 2021a). To date, the content of most social influencer trainings has focused primarily on providing social influencers with knowledge about the promoted health behaviors (St. Pierre et al., 2021). Behavioral change models assume that more determinants than having the capability or the necessary knowledge are needed to engage in the health behavior and that motivation also plays a central role (Michie et al., 2011; Deci and Ryan, 2012). Therefore, health intervention developers should consider the social influencers' motivation to engage in the health behavior themselves and their motivation to promote the desired health behavior within their social networks. To address this, we propose to apply principles of a prominent theory of motivation in health behavior, called self-determination theory, in the training of the social influencers.

According to self-determination theory (Deci and Ryan, 2012), being intrinsically motivated depends on the degree to which individuals perceive the satisfaction of three psychological needs: autonomy (the sense of ownership and choice), competence (the sense of being capable and effective), and relatedness [the sense of being connected and caring for others (Deci and Ryan, 2000; Ryan and Deci, 2017)]. These psychological needs can be satisfied by creating a context in which, for example, individuals' perspectives are acknowledged, rationales are provided that are personally meaningful to them, choices are offered, and support is provided to strengthen their existing skills, all while minimizing pressure and control (Deci and Ryan, 2000; Vansteenkiste et al., 2004). Several studies have shown that applying these types of motivational techniques contributes to promoting intrinsic motivation and desired behavioral outcomes over time [for a review, see Gillison et al. (2019), Teixeira et al. (2020)]. Therefore, to enhance the intrinsic motivation of social influencers in health interventions, techniques from self-determination theory can be applied in the training to satisfy their needs for autonomy, competence, and relatedness. In a recent study, we applied techniques from self-determination theory in the design and delivery of a health intervention with social influencers. The study showed that social influencers' intrinsic motivation for the desired health behavior increased, and that they felt supported in promoting the health behavior within their social networks (Smit et al., 2021b). In the next section we provide concrete examples of techniques that can be applied in health interventions to motivate social influencers to engage in health interventions. To do so, we will utilize insights from self-determination theory and our empirical experience with implementing health behavior interventions with social influencers.

## Techniques for Motivating Social Influencers

### Personal Approach

Today's youth spend a lot of time online (Reid Chassiakos et al., 2016; Valkenburg and Piotrowski, 2017) and are often permanently connected to their online social networks through social media platforms (Boyd, 2014). Many health intervention developers tend to conduct health interventions with social influencers entirely online, because this is a low-cost and less time-consuming method (Bell et al., 2017). However, we recommend against an online-only approach for the training or briefing. A recent health intervention in which social influencers were trained entirely online and where there were no face-to-face interactions with the social influencers did not find an effect of the intervention on youth's health behaviors (van Woudenberg et al., 2018). The researchers argued that, compared to health interventions offering face-to-face interactions, this less personal approach resulted in lower social influencer engagement in the health intervention. The relationship between the intervention developer and the influencer has been shown to be an important factor in determining the success of an intervention (Santiago and Castelo, 2020). Through face-to-face interactions, social influencers experience that there is personal involvement and care from the intervention developers and that time is taken for them which contributes to their sense of relatedness (Gillison et al., 2019). Providing opportunities for ongoing support during the delivery of the health intervention to social influencers is also a technique that corresponds to their need for relatedness (Deci and Ryan, 2000; Gillison et al., 2019; Teixeira et al., 2020). A systematic review indicated that providing follow-up sessions and/or having regular contact to provide support are important factors to include when implementing health interventions involving social influencers (St. Pierre et al., 2021). All of these techniques ultimately increase the intrinsic motivation of social influencers to engage in health interventions (Deci and Ryan, 2000; Gillison et al., 2019).

### Non-controlling Language

The way in which the training or briefing on health behaviors is communicated to social influencers is important to ensure that they feel connected to and internalize the information conveyed (Pope et al., 2018). When non-controlling and informational language is applied, individuals are more likely to perceive choice and collaboration, which responds to their need for autonomy (Deci and Ryan, 2000; Gillison et al., 2019; Teixeira et al., 2020). In contrast, a controlling approach has been found to be experienced as external pressure for individuals, which could result in social influencers feeling that performing the behavior is not their own choice (Weinstein et al., 2020). These feelings of external pressure can evoke reactance whereby they disregard the health behavior or do the opposite (van Petegem et al., 2015). Research has shown that when trainers used non-controlling and informational language in their communication with social influencers, they felt that they were offered choices, encouraged to ask questions, heard and understood, which promoted their intrinsic motivation to engage in the behavior (Sebire et al., 2019; Smit et al., 2021b).

### Collaborative Activities

A personal approach allows for interactive elements to be added to the training, which is an important contributor to ensuring that the training is well received by social influencers (St. Pierre et al., 2021). Games, group discussion, and collaboration are important training elements to increase the enjoyment of social influencers for the desired behavior (Sebire et al., 2019). Collaborative activities can be incorporated into training or briefing in two ways. First, opportunities can be created to perform the task of promoting the health behavior as a group. Facilitating cooperative peer-to-peer activities within the group contributes to their sense of relatedness which may increase their intrinsic motivation to promote the behavior (Deci and Ryan, 2000; Gillison et al., 2019). In our recent study, social influencers indicated that collaborating on a “secret mission” was the most enjoyable aspect of the health intervention (Smit et al., 2021b). Second, collaborative activities can be applied by encouraging them to share with others their perspectives on the behavior during the training. These perspectives can inform their personal choices, tapping into their feelings of autonomy. Consequently, this may increases their motivation to engage in the behavior (Deci and Ryan, 2000; Teixeira et al., 2020).

### Personal Meaningful Rationales

Providing meaningful rationales has been found to be an effective technique to apply in health interventions (Dusseldorp et al., 2014). Research has shown that even in a boring task, meaningful rationales can lead to internalization (Deci et al., 1994). Encouraging social influencers to identify rationales for health behaviors that are personally meaningful or valuable to them addresses their sense of autonomy, which forms the basis for their intrinsic motivation (Deci and Ryan, 2000; Gillison et al., 2019; Teixeira et al., 2020). Providing social influencers with rationales to engage in and promote the health behaviors devised by intervention developers may feel to them as being externally imposed (Martela et al., 2021). Instead, rationales can be more persuasive if they align with the perceptions of the social influencers. In this regard, it is important that the rationales are cocreated with the social influencer and that their vision is taken into account, aligning with their need for autonomy. In our recent study, social influencers indicated that devising rationales, using a word web, on the importance of the health behavior was one of the most enjoyable aspects of the training. Moreover, they shared these devised rationales when communicating the importance of the health behavior in their social network (Smit et al., 2021b).

### Strengthen Existing and Build New Skills

Prompting social influencers to identify potential barriers when engaging in and promoting health behaviors and exploring ways to overcome them increases their self-confidence and reinforces their existing skills, relating to their need for competence (Deci and Ryan, 2000; Gillison et al., 2019; Teixeira et al., 2020). This can be achieved by using self-persuasion techniques (Aronson, 1999); for example, by placing them in a situation where they can devise their own arguments to convince themselves how to adhere to or change the behavior (Miller and Wozniak, 2001). Devising reasons and ways how they could engage in and promote the health behaviors within their social network creates an opportunity for them to learn new skills. In addition, the training or briefing can also focus on supporting and informing them about the use of possible strategies to promote the health behavior. These techniques boost their self-confidence, reduce feelings of failure, and promote their sense of competence (Deci and Ryan, 2000; Gillison et al., 2019). Research has shown the importance of having the opportunity to practice promoting the health behavior, for example through role playing (St. Pierre et al., 2021). As mentioned earlier, when providing information about the strategies they can use, it is important that social influencers experience freedom of choice. This promotes ownership which contributes to their sense of autonomy and consequently increases their intrinsic motivation (Deci and Ryan, 2000; Gillison et al., 2019; Teixeira et al., 2020).

Altogether, the use of these techniques can increase the intrinsic motivation of social influencers (Deci and Ryan, 2000; Gillison et al., 2019; Teixeira et al., 2020), which can ensure that they create authentic and original content that is perceived as attractive and credible by the individuals in their social networks (Casaló et al., 2020). Providing and encouraging creative freedom is found to be an important aspect for effective interventions with social influencers (Santiago and Castelo, 2020). However, our research showed that when social influencers are given full freedom, some influencers tend to use promotional strategies that can be considered as controlling, for example, by making it a challenge and offering rewards in return (Smit et al., 2021b). This does not spark the intrinsic motivation of the individuals in their social network (Cameron and Pierce, 1994). Therefore, in addition to intrinsically motivating social influencers, it is also important to educate them about promoting health behaviors in a way that promotes the intrinsic motivation of the individuals in their social network.

## Conclusion

In conclusion, our perspective is that health behavior interventions involving social influencers should have an extensive focus on the intrinsic motivation of social influencers. This can be achieved by integrating insights and techniques from self-determination theory to enhance the intrinsic motivation of social influencers through the satisfaction of their psychological needs. Enhancing the intrinsic motivation of social influencers in health interventions makes them more likely to engage, and thus promote the behavior in a more authentic, original, and credible way. In this paper, we presented a set of techniques that can be applied to intrinsically motivate social influencers. We believe that the needs of social influencers could be more effectively addressed by a cocreation approach in which they are involved throughout the development and implementation of the intervention content (Anselma et al., 2019). A cocreation approach underlines feelings of autonomy, competence, and relatedness among social influencers because they are listened to and have freedom of choice in their actions and behaviors. We acknowledge that besides the motivation of social influencers, having the opportunity to engage in the health behavior and the ability to promote the behavior are also important determinants for behavior change (Michie et al., 2011). In addition to the motivation of the social influencers, it is also important to focus on the intrinsic motivation of targeted individuals in their social networks, especially for the long-term effect of the health behavior intervention (Silva et al., 2011; Smit et al., 2018). Future research should therefore focus on supporting the social influencers in creating a social climate that promotes the motivation for health behaviors of the individuals in their social network.

## Author Contributions

CS conceptualized the ideas and wrote the first draft of the article. KB, MB, and RL contributed to the conceptualization. KB involved in writing of the article. RL contributed to the final version of the article. MB edited the article. All authors contributed to the article and approved the submitted version.

## Funding

This publication is part of the project SocialMovez: Effective and responsible health campaigns for adolescents using online social networks (with project number VI.C.181.045) of the Vici Talent Program, which is financed by the Dutch Research Council (NWO).

## Conflict of Interest

The authors declare that the research was conducted in the absence of any commercial or financial relationships that could be construed as a potential conflict of interest.

## Publisher's Note

All claims expressed in this article are solely those of the authors and do not necessarily represent those of their affiliated organizations, or those of the publisher, the editors and the reviewers. Any product that may be evaluated in this article, or claim that may be made by its manufacturer, is not guaranteed or endorsed by the publisher.
